# SMARTphone-based, early cardiac REHABilitation in patients with acute coronary syndromes [SMART-REHAB Trial]: a randomized controlled trial protocol

**DOI:** 10.1186/s12872-016-0356-6

**Published:** 2016-09-05

**Authors:** Matias B. Yudi, David J. Clark, David Tsang, Michael Jelinek, Katie Kalten, Subodh Joshi, Khoa Phan, Arthur Nasis, John Amerena, Sandeep Arunothayaraj, Chris Reid, Omar Farouque

**Affiliations:** 1 Department of Cardiology, Austin Health, Melbourne, Australia; 2 Department of Medicine, University of Melbourne, Melbourne, Australia; 3 Department of Cardiology, Western Health, Melbourne, Australia; 4 Department of Cardiology, St Vincent’s Hospital, Melbourne, Australia; 5 Department of Heart and Mind, Australian Catholic University, Melbourne, Australia; 6 Monash Heart, Monash Health, Melbourne, Australia; 7 Department of Cardiology, Royal Melbourne Hospital, Melbourne, Australia; 8 Department of Cardiology, Barwon Health, Geelong, Australia; 9 School of Public Health, Curtin University, Perth, Western Australia Australia

**Keywords:** Cardiac rehabilitation, Secondary prevention, Acute coronary syndromes, mhealth, Smartphone application

## Abstract

**Background:**

There are well-documented treatment gaps in secondary prevention of coronary heart disease and no clear guidelines to assist early physical activity after acute coronary syndromes (ACS). Smartphone technology may provide an innovative platform to close these gaps. This paper describes the study design of a randomized controlled trial assessing whether a smartphone-based secondary prevention program can facilitate early physical activity and improve cardiovascular health in patients with ACS.

**Methods:**

We have developed a multi-faceted, patient-centred smartphone-based secondary prevention program emphasizing early physical activity with a graduated walking program initiated on discharge from ACS admission. The program incorporates; physical activity tracking through the smartphone’s accelerometer with interactive feedback and goal setting; a dynamic dashboard to review and optimize cardiovascular risk factors; educational messages delivered twice weekly; a photographic food diary; pharmacotherapy review; and support through a short message service. The primary endpoint of the trial is change in exercise capacity, as measured by the change in six-minute walk test distance at 8-weeks when compared to baseline. Secondary endpoints include improvements in cardiovascular risk factor status, psychological well-being and quality of life, medication adherence, uptake of cardiac rehabilitation and re-hospitalizations.

**Discussion:**

This randomized controlled trial will use a smartphone-phone based secondary prevention program to emphasize early physical activity post-ACS. It will provide evidence regarding the feasibility and utility of this innovative platform in closing the treatment gaps in secondary prevention.

**Trial registration:**

The trial was retrospectively registered in the Australian New Zealand Clinical Trials Registry (ANZCTR) on April 4, 2016. The registration number is ACTRN12616000426482.

## Background

Coronary heart disease (CHD) is the leading cause of mortality and morbidity in the world [1]. In 2012 CHD accounted for approximately 7.4 million lives, an increase of 23 % (1.4 million) from CHD deaths in 2000 [1]. Patients at highest risk of premature death, myocardial infarction and re-hospitalization are those with known CHD [2]. Consequently international guidelines strongly advocate proven secondary prevention strategies such evidence-based pharmacological therapy, cardiovascular risk factor optimization, cardiac rehabilitation and adherence to diet and physical activity recommendations [3].

Cardiac rehabilitation provides a comprehensive secondary prevention framework based on extensive scientific evidence. A recent Cochrane review revealed cardiac rehabilitation was associated with a 13 % decrease in all-cause mortality and a 26 % decrease in cardiovascular mortality [4]. This result is consistent with several other meta-analyses [5–7].

However, there is poor translation of scientific evidence into clinical practice. Cardiac rehabilitation services are underutilized as highlighted by low referral rates and even lower completion rates [8–12]. In addition, utilization of recommended pharmacological therapy and compliance with dietary and physical activity guidelines is suboptimal [13–16]. Lastly, outpatient cardiac rehabilitation often starts weeks to months after the index event, even with the utilization of new technology [17]. This delay creates a missed opportunity to immediately reinforce the importance of physical activity and lifestyle changes. Furthermore, the delay in cardiac rehabilitation has been associated with delayed resumption of work [18].

The drawbacks of conventional cardiac rehabilitation have resulted in the development of innovative models of care. In the seminal paper by Vale et al, regular personal coaching via telephone has been shown to be effective in reducing total cholesterol levels and other cardiovascular risk factors [19]. Systematic reviews and meta-analyses have shown telehealth interventions for secondary prevention, limited to telephone calls, internet and videoconferencing technologies, offer an effective alternative model of secondary prevention care [20, 21].

Most recently, the Tobacco, Exercise and Diet Messages (TEXT ME) study showed a lifestyle-focused text-messaging service lowered LDL-cholesterol level, and blood pressure while increasing smoking cessation rates and physical activity in patients with known CHD [22].

Smartphone technology is an advance on previous telehealth and text-message based technologies. It has the potential to revolutionize the landscape of secondary prevention as it can provide a platform for a patient-centred program with the capacity to incorporate education, real-time feedback, motivation, reminders and support. Furthermore, it is a tool that can be used to monitor diet, physical activity, medications and cardiovascular risk factor parameters. Lastly and most importantly, a smartphone-based program can be started immediately, delivered from anywhere at any time and for extended periods of time.

Four studies have been published incorporating smartphone technology for the delivery of cardiac rehabilitation. Worringham et al. assessed a walking-based cardiac rehabilitation program with single-lead ECG, heart rate and GPS-based speed and location transmitted via smartphone for real-time monitoring by a qualified exercise scientist. [23] Six patients were evaluated in 134 exercise sessions with improvements in exercise capacity (improvement in 6-minute walk test from 524 m to 637 m, *p* < 0.01) and health status measured by SF-36 form. Korzeniowska-Kubacka et al. assessed a mobile device with preprogrammed exercise training sessions with audio and visual clues and a 3-lead ECG monitor [24]. In this non-randomized trial, 30 patients were assigned to the mobile device intervention with 10 supervised exercise sessions and 14 sessions with the mobile device at home. The control group of 32 patients had 24 supervised exercise sessions. There was in improvement in exercise capacity in the mobile arm (*p* < 0.05) but no difference in blood pressure. Blasco et al. assessed a web-based telemonitoring system that provided patients with self-measurement devices and connected them to a cardiologist who accessed the data and sent recommendations via short message service (SMS) [25]. In this single-blinded, randomized controlled trial of 203 acute coronary syndrome survivors, patients in the telemonitoring group were more likely to improve at least one risk factor (RR 1.4, 95 % CI 1.1–1.7) and achieve targets for BP (62.1 % vs 42.9 %, *p* = 0.01) and HbA1c (86.4 % vs 54.2 %, *p* = 0.02). Varnfield et al. compared the effectiveness of smartphone-based cardiac rehabilitation versus traditional care on cardiac rehabilitation uptake, adherence and completion in a randomized controlled trial [17]. 120 patients were randomized to the two groups for a 6-week cardiac rehabilitation program followed by a 6-month self-maintenance period. The smartphone based model included health and exercise monitoring, motivational and educational material delivery and weekly mentoring consultations. The trial met its primary end point with the smartphone based rehabilitation program showing significantly higher uptake (94 % vs. 68 %, *P* <0.05) and completion (80 % vs. 47 %, *p* <0.05).

The exponential growth and availability of smartphone technology provides a novel opportunity to optimize secondary prevention of coronary heart disease [26]. However, further studies are needed to examine the value of this approach and to demonstrate improvements in objective outcomes.

### AIM

The aim of this study is to assess the impact of a smartphone-based secondary prevention program (SSPP), initiated on discharge in patients with acute coronary syndromes, on exercise capacity, cardiovascular risk factors, quality of life and mood.

#### Hypothesis

The primary hypothesis is that a SSPP would improve exercise capacity, as measured by a 6-minute walk test, compared to standard care. Secondary hypotheses are: A SSPP will result in improvement in cardiac depression and quality of life scores as well as in cardiovascular risk factors (lipid profile, blood pressure, HbA1C, anthropometry) and secondary prevention pharmacotherapy use between the groups.

## Methods/design

**Fig. 1:**
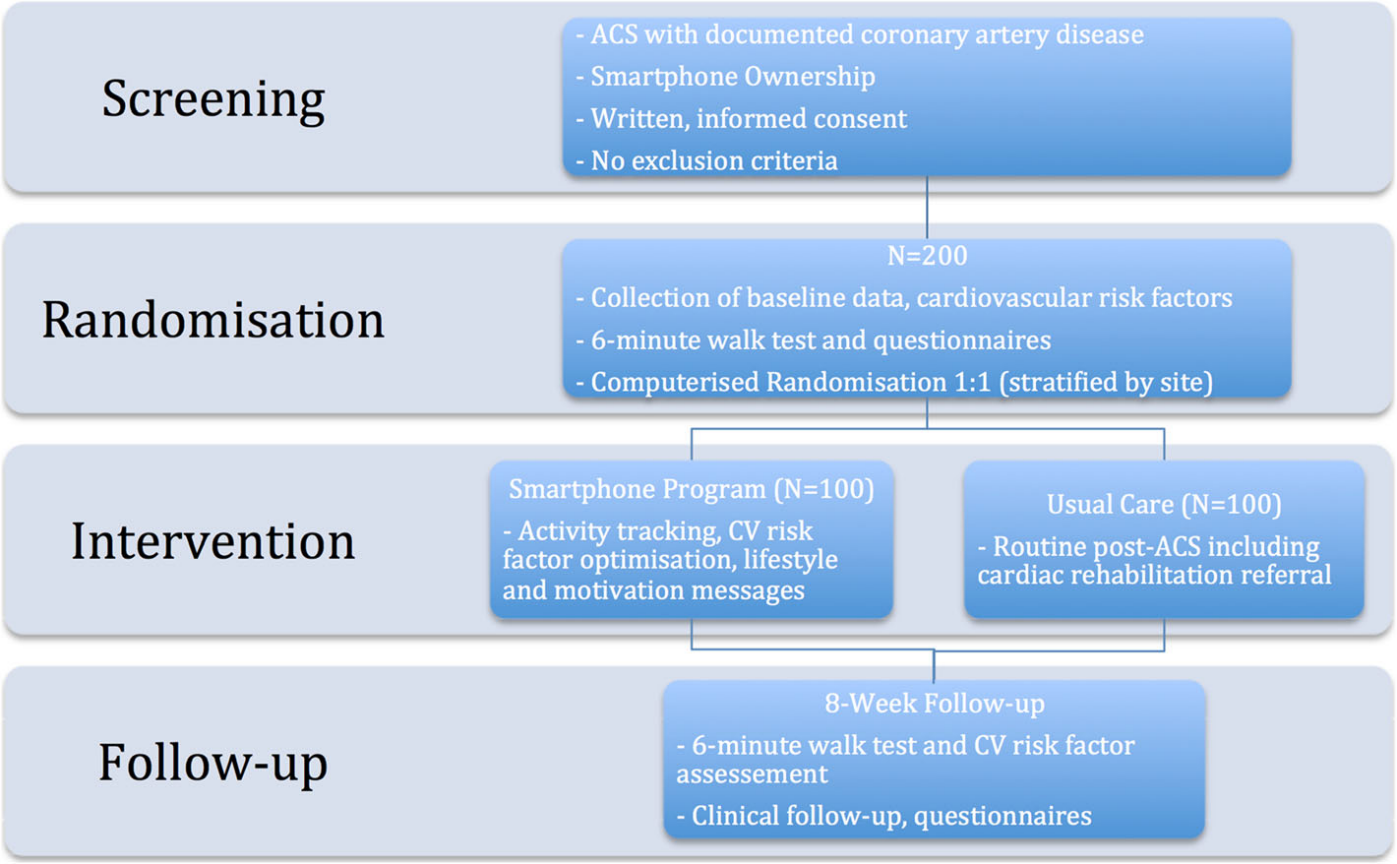
Randomized Control Trial Design and Flow

The study design is single-blinded, two-arm, parallel, randomized control trial. The study design is displayed in Fig. 1. The protocol conforms to the SPIRIT 2013 statement and the intervention is described in accordance with the CONSORT-EHEALTH checklist. [27–30]

**Fig. 2:**
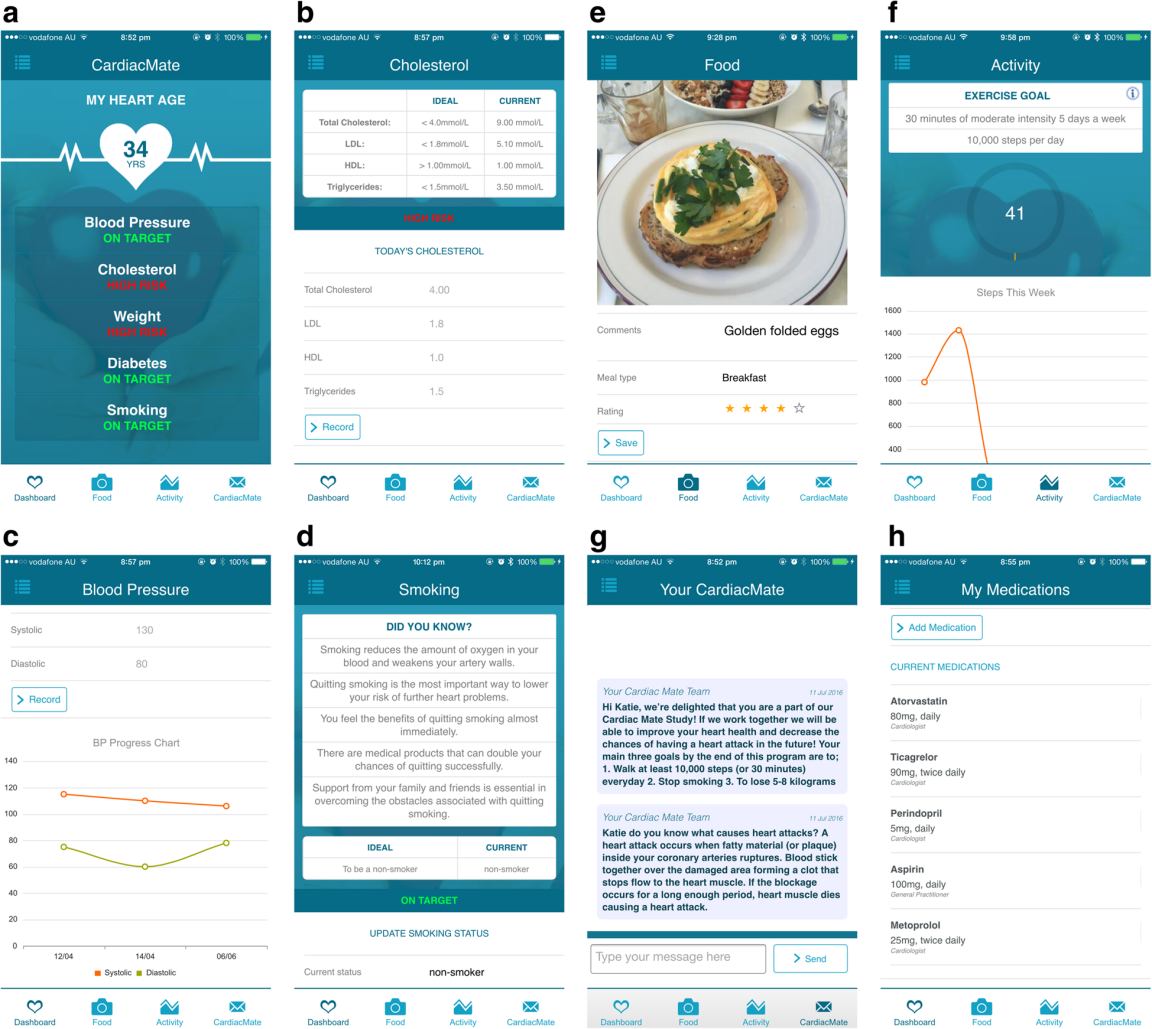
Smartphone application interface depicting interactive dashboard (**a**), cholesterol page (**b**), blood pressure progress chart (**c**), smoking education page (**d**), photographic food diary (**e**), activity tracking page (**f**), individualized messaging service (**g**) and medication page (**h**)

### Eligibility and recruitment

Patient over the age of 18 with a diagnosis of an acute coronary syndrome and documented coronary artery disease on coronary angiography (coronary artery stenosis >50 %), who are treated either medically or with percutaneous coronary intervention will be eligible for inclusion. The other major inclusion criteria will be personal ownership of a smartphone.

Exclusion criteria include untreated ventricular tachycardia, severe heart failure, significant residual coronary artery disease requiring revascularization, treatment with coronary artery bypass surgery, coexisting disease with a life expectancy less than 1 year and/or significant exercise limitations for reasons other than CHD.

Recruitment will occur during hospital admission at six major university affiliated teaching hospitals in Victoria, Australia. All patients admitted with acute coronary syndromes will be screened and those meeting the entry criteria will be selected. Potential participants will receive study information and informed consent will be obtained.

### Sample size calculation

A sample size of 152 participants (76 per group) will be required to detect a minimal clinically important difference of 25 m for the six-minute walk test with 80 % power at 5 % level of significance [31, 32]. To account for an estimated 20 % loss to follow-up, 100 patients in each arm will be recruited.

### Ethics approval

The Austin Health Human Research Ethics Committee granted approval for the trial (HREC/14/AUSTIN/128).

### Randomization and blinding

Eligible patients will be randomized in a 1:1 fashion to smartphone-based secondary prevention program or usual care through a computerized randomization program. Randomization will be stratified by study site and be overseen by the Centre of Cardiovascular Research and Education in Therapeutics at Monash University; an independent research body within the School of Public Health and Preventive Medicine at Monash University, Melbourne, Australia. Baseline assessment, including the 6-minute walk test, will occur prior to randomization. Assessors of the primary outcome are blind to treatment allocation; however participants are not blinded.

### Intervention

The smartphone-based secondary prevention program will be delivered over 8 weeks starting at time of discharge from hospital through a smartphone application (app). Participants in the smartphone intervention cohort will download the intervention application (“app”) into their smartphone. They will then receive education on how to use the app. The SSPP is a multi-faceted intervention with particular emphasis on early physical activity. The app provides a platform to deliver a comprehensive secondary prevention program (Fig. 2). Participants can log off the app at any time effectively not undertaking the SSPP. The different components of the SSPP are discussed in detail below.

#### Exercise prescription

Patients will have access to real-time feedback of their activity levels through the app’s activity tracker which links to the smartphone’s accelerometer feature. Patients will be able to monitor the number of steps taken and the distance walked. Patients will undertake a graded walking program increasing in time and intensity over the 8 weeks. Their aim will be to achieve 30 min of walking a day for at least 5 days every week, in accordance with guideline recommendations [3]. Patients will be encouraged to progress to moderate intensity activity, defined as a noticeable increase in depth and rate of breathing while still being able to talk comfortably, within 2 weeks.

#### Dynamic tracking of cardiovascular risk factors

Patients will have an interactive personal dashboard highlighting the status of their cardiovascular risk factors. Blood pressure, cholesterol levels, glucose levels, weight, smoking status will be graded depending whether they are at recommended target levels [3]. Patients will record this information in the app prior to discharge and will be able to update this at anytime.

#### Dietary habits

Dietary habits will be tracked through the app via access to the phone’s inbuilt camera. Patients will be encouraged to photograph the food they consume. They will have capabilities to rate and comment on the food they are communing based on their perception of an ideal diet as per guidelines.

#### Cardiac education

Two educational messages per week will be delivered to the participants as they progress through the 8-week SSPP.

#### Cardiac medication list

Patients will input their discharge medications into the app prior to leaving hospital. The medication list will be reviewed to ensure that appropriate evidence-based pharmacotherapy (dual antiplatelet therapy, statin, angiotensin converting enzyme inhibitor/angiotensin receptor blocker, beta-blocker) has been prescribed. If a particular class of drug has not been prescribed, the patient will be notified through the app and encouraged to discuss this further with their cardiologist or general practitioner. Participants will be able to edit their medication list whenever a change is made.

#### Interactive and personalized feedback

Patients will receive five personalized messages per week via the app messaging service. Two of the messages will be educational and the remaining three messages provide goal setting and feedback on physical activity levels and diet. The behavioural change strategies implemented are based on social cognitive theory and have been previously successfully applied in mobile health technologies [33]. There is emphasis on increasing confidence and motivation to exercise while overcoming barriers to being physically active. Furthermore, there is clear goal setting prescribed every week regarding physical activity and diet. Feedback is given based on exercise levels (automated) and diet (personalized). The app allows the patient to analyze their own physical activity and assess their progress towards their individualized goal. Table 1 shows sample messages.

**Table 1:** Sample Messages

Physical Activity • Hi [name], walking is a great type of exercise for people with coronary heart disease – I see you did xxxx steps yesterday. Over the next few days, aim to walk at least 500 steps more a day! • Hi [name], well done on your activity over the past week! For this week, your goal is to walk for at least 20 min a day. Given your best last week was xxxx steps, this week you should reach xxxx steps a day! • Hi [name], well done on reaching xxxx steps last week! This week, your aim is to walk 30 min a day everyday. Make it a routine as they benefits will last a lifetime!
Dietary Habits • Hi [name], diet is very important in reducing the risk of future heart attacks. Your aim for the week is to try and eat 5 serving of vegetables everyday. Make sure you take photos of your meals so you are accountable for what you are eating! • Hi [name], making small, healthy changes in the food and drinks that you have can make a big difference. Over the next week can you try and eliminate all soft drinks and junk food. Keep us posted with your electronic food diary! • Hi [name], did you know that oily fish contains polyunsaturated fat that helps to lower your cholesterol? You should try and have 2–3 serves of fish each week.
Educational • [Name], do you know what causes heart attacks? A heart attack occurs when fatty material (or plaque) inside your coronary arteries ruptures. Blood sticks together over the damaged area forming a clot that stops flow to the heart muscle. If the blockage occurs for a long enough period, heart muscle dies causing a heart attack. • [Name], did you know it is essential to take your prescribed medications? They will reduce your risk of further heart attacks, strokes and premature death. You should be taking 5 medications for your heart. Check with your doctor if you have any questions about them or if need a new script – suddenly stopping your medications can place you at high risk of further heart attacks. • [Name], did you know most people can have sex soon after a heart attack? If you can walk up two flights of stairs without getting breathless or having chest pain, you are probably well enough to have sex. If you have any concerns, don’t hesitate to contact your doctor.

#### Support

The messaging service also allows the patient to initiate contact if they have any questions regarding their cardiac condition or rehabilitation process. Replies will be made within one business day.

### Standard care

Regardless of the treatment arm, all patients will receive standard of care treatment post-ACS. This includes inpatient cardiology review, pre-discharge planning, formal communication with general practitioner through a discharge summary letter, referral to cardiac rehabilitation, promotion of self care and a chest pain action plan.

### Outcome assessment

Participants will be evaluated at baseline, prior to discharge from hospital, and at 8 weeks, as an outpatient. The initial assessment will include explanation of study protocol and signing of consent forms. Patients assigned to SSPP will undertake app training to ensure they can maximize their experience. Baseline evaluation will include measurement and assessment of height, weight, waist circumference, resting heart rate and blood pressure, smoking status, fasting cholesterol and glucose levels and exercise capacity measured by a six-minute walk test.

#### Primary outcome

The primary outcome is change in six-minute walk distance that will be measured in standardized fashion [34]. The initial test will be undertaken prior to hospital discharge and this will be repeated at 8 weeks as an outpatient, without monitoring.

#### Secondary outcomes

In general, all secondary outcomes will be measured at baseline and at 8-week follow-up. Table 2 summarises the secondary outcomes of our study.

**Table 2:** Primary and secondary endpoints

Primary Outcome • Change in six-minute walk test distance
Secondary Outcomes • Fasting lipid levels (TC, LDL-C, HDL-C, TG) • Fasting glucose • Resting blood pressure • Weight and body mass index • Waist circumference • Smoking status (self-reported) • Uptake, Adherence and Completion of cardiac rehabilitation • Major adverse cardiovascular events • Cardiovascular hospital re-admissions • Depression assessment (CDS and DS-SF questionnaires) • Anxiety assessment (HADS questionnaire) • Quality of life assessment (SF-36 questionnaire) • Health outcome assessment (EQ-5D questionnaire) • Time to return to work • Secondary prevention pharmacotherapy use (self-reported) • Six minute walk test distance at 8-weeks

Body mass index will be derived from the weight (kg) divided by height (m) squared. Waist circumference will be measured using an anthropometric tape placed around the participant’s waist at the level of the umbilicus. An electronic sphygmomanometer with pulse oximetry will be used to assess the systolic and diastolic blood pressure as well as resting heart rate.

Fasting glucose and cholesterol levels will be measured after 12 h of fasting. It will be analyzed at the pathology department of the corresponding hospital in standard fashion.

Smoking status, medication compliance and current medication list will be assessed via self-reporting at the 8-week assessment. The date the participant returns to work, if applicable, will also be collected to assess the time to resumption of work after acute coronary syndromes.

Uptake, adherence and completion of a cardiac rehabilitation program will be assessed. Uptake is defined as attending at least one exercise session for traditional cardiac rehabilitation or uploading 1 day’s physical activity in the SSPP. Adherence is defined as attending 4 weeks’ or uploading 12-days of activity over 4 weeks of physical activity. Completion is defined as showing adherence to cardiac rehabilitation and attending the 8-week assessment session.

Major adverse cardiovascular events (MACE) will be recorded. MACE is defined as the composite of death, myocardial infarction, stroke and unplanned revascularization.

Assessment for depression will be undertaken with the Cardiac Depression Scale (CDS) and Depression Scale – Short Form (DS-SF) [35]. Assessment of anxiety will be undertaken with the use of the Hospital Anxiety and Depression Scale (HADS). Change in quality of life will be assessed by the SF-36 questionnaire [36].

The EQ5D will be used to obtain a single preference index for calculation of Quality Adjusted Life Years (QALY) to assess cost per QALY for comparison to standard care. Health care utilization will be recorded for adverse events including presentation to hospital and/or general practitioner for symptoms of coronary heart disease or heart failure.

### Statistical analysis

Statistical analyses will be performed using SPSS (IBM, USA). Baseline characteristics will be summarized using descriptive statistics. Continuous variables will be described as mean and standard deviation and be compared using Student’s *t* test. Categorical variables will be described as frequencies and percentages and compared using Fisher’s exact or chi-square tests as appropriate. Treatment evaluation will be performed on an intention to treat (ITT) analysis.

## Discussion

This novel study aims to evaluate the impact of a smartphone based secondary prevention program on functional capacity of patients with acute coronary syndromes. The benefit of comprehensive secondary prevention programs incorporating physical activity is well recognised. Smartphone technology could hold the key to bridging the well documented gap between scientific evidence and clinical practice.

This randomised clinical trial also aims to build the scientific base supporting alternative models of secondary prevention care in patients post acute myocardial infarction. Of particular note, is the initiation of this program as soon as the patient is discharged from hospital post their index event. This is a crucial period of recovery and immediate initiation of smartphone based secondary prevention program may facilitate early physical activity and return to normal daily function, including resumption of work.

The primary endpoint aims to objectively assess improvement in physical activity through the use of the six-minute walk test. Near maximal exercise testing is safe in patients with uncomplicated myocardial infarction prior to discharge and thus early mobilisation should be encouraged [37, 38]. The superiority trial design is suitable to detect a minimal clinically important difference in 6-minute walk distance [31]. It is postulated the improvement will be due to earlier initiation of an exercise program and increase in uptake and adherence with cardiac rehabiliation.
